# The calcium-sensing receptor regulates parathyroid hormone gene expression in transfected HEK293 cells

**DOI:** 10.1186/1741-7007-7-17

**Published:** 2009-04-27

**Authors:** Hillel Galitzer, Vardit Lavi-Moshayoff, Morris Nechama, Tomer Meir, Justin Silver, Tally Naveh-Many

**Affiliations:** 1 Minerva Center for Calcium and Bone Metabolism, Nephrology Services, Hadassah-Hebrew University Medical Center, Jerusalem, Israel

## Abstract

**Background:**

The parathyroid calcium receptor determines parathyroid hormone secretion and the response of parathyroid hormone gene expression to serum Ca^2+ ^in the parathyroid gland. Serum Ca^2+ ^regulates parathyroid hormone gene expression *in vivo *post-transcriptionally affecting parathyroid hormone mRNA stability through the interaction of *trans*-acting proteins to a defined *cis *element in the parathyroid hormone mRNA 3'-untranslated region. These parathyroid hormone mRNA binding proteins include AUF1 which stabilizes and KSRP which destabilizes the parathyroid hormone mRNA. There is no parathyroid cell line; therefore, we developed a parathyroid engineered cell using expression vectors for the full-length human parathyroid hormone gene and the human calcium receptor.

**Results:**

Co-transfection of the human calcium receptor and the human parathyroid hormone plasmid into HEK293 cells decreased parathyroid hormone mRNA levels and secreted parathyroid hormone compared with cells that do not express the calcium receptor. The decreased parathyroid hormone mRNA correlated with decreased parathyroid hormone mRNA stability *in vitro*, which was dependent upon the 3'-UTR *cis *element. Moreover, parathyroid hormone gene expression was regulated by Ca^2+ ^and the calcimimetic R568, in cells co-transfected with the calcium receptor but not in cells without the calcium receptor. RNA immunoprecipitation analysis in calcium receptor-transfected cells showed increased KSRP-parathyroid hormone mRNA binding and decreased binding to AUF1. The calcium receptor led to post-translational modifications in AUF1 as occurs in the parathyroid *in vivo *after activation of the calcium receptor.

**Conclusion:**

The expression of the calcium receptor is sufficient to confer the regulation of parathyroid hormone gene expression to these heterologous cells. The calcium receptor decreases parathyroid hormone gene expression in these engineered cells through the parathyroid hormone mRNA 3'-UTR *cis *element and the balanced interactions of the *trans*-acting factors KSRP and AUF1 with parathyroid hormone mRNA, as *in vivo *in the parathyroid. This is the first demonstration that the calcium receptor can regulate parathyroid hormone gene expression in heterologous cells.

## Background

Parathyroid hormone (PTH) regulates calcium homeostasis and bone metabolism. Changes in extracellular Ca^2+ ^([Ca^2+^]_o_) are sensed by the parathyroid G-protein coupled calcium receptor (CaR) [1]. The CaR determines the response of the parathyroid to [Ca^2+^]_o _at the levels of PTH secretion, PTH gene expression and parathyroid cell proliferation [2,3]. Increased [Ca^2+^]_o _activates the CaR, resulting in a G-protein-dependent activation of PLC, PLA2 and PLD [4]. This results in decreased PTH secretion and parathyroid cell proliferation. Calcimimetics bind transmembrane (TM) 6 and TM7 of the CaR to allosterically alter the conformation of the CaR [5,6]. The calcimimetic R568 decreases PTH secretion, PTH mRNA levels and parathyroid cell proliferation [7,8]. Genetic deletion of G_q/11 _specifically in the parathyroid leads to severe hyperparathyroidism (HPT) [9]. Similarly, *CaR*^-/- ^mice are not viable due to the severe HPT [10] and can be rescued by mating with *PTH*^-/- ^or *GCM2*^-/- ^mice, where PTH is either absent or markedly reduced [11,12]. Therefore, the CaR and its signal transduction are central to parathyroid physiology and the maintenance of a normal serum PTH and intact Ca^2+ ^homeostasis.

Low serum Ca^2+ ^and chronic kidney disease lead to secondary hyperparathyroidism which is characterized by increased PTH mRNA levels in experimental models [13]. The increased PTH mRNA *in vivo *is post-transcriptional and is mediated by the interaction of *trans*-acting proteins to a defined *cis*-acting AU-rich element (ARE) in the PTH mRNA 3'-untranslated region (UTR) [14-16]. A 26-nucleotide sequence within the ARE is conserved among species and is both necessary and sufficient for protein binding and the regulation of PTH mRNA stability by dietary calcium or phosphorus depletion [16,17]. AU-rich binding factor 1 (AUF1) and Upstream of N-*ras *(Unr) are PTH mRNA *trans*-acting proteins that stabilize PTH mRNA [18,19]. The binding of these proteins to the PTH mRNA 3'-UTR is regulated in the parathyroid by chronic hypocalcemia, hypophosphatemia and experimental kidney failure as well as by the calcimimetic R568 [7,15,16]. We have recently identified the decay-promoting protein KSRP (KH domain splicing regulatory protein) as an additional PTH mRNA 3'-UTR binding protein that determines PTH mRNA stability in transfected cells [20]. KSRP-PTH mRNA interaction is increased in parathyroids from hypophosphatemic rats, where PTH mRNA is unstable, and decreased in parathyroids from hypocalcemic and experimental renal failure rats, where the PTH mRNA is more stable. The balanced interaction of PTH mRNA with AUF1 and KSRP determines PTH mRNA half-life and levels and hence serum PTH levels [20].

There is no parathyroid cell line and the signal transduction pathway whereby [Ca^2+^]_o _regulates PTH secretion has been characterized by using bovine parathyroid cells in suspension and rat parathyroid organ cultures [21,22]. Parathyroid hormone gene expression and its regulation in cells *in vitro *have been studied in primary cultures of bovine parathyroids [23,24]. HEK293 cells transfected with the CaR faithfully maintain an intact signal transduction after the stimulus of a high [Ca^2+^]_o _to activate the mitogen-activated protein kinase (MAPK) pathway [25,26]. We have now utilized this transfected heterologous cell system to study the mechanisms whereby the CaR regulates PTH gene expression. As the regulation of PTH gene expression by [Ca^2+^]_o _is predominantly post-transcriptional, we studied PTH mRNA stability in a system that was independent of any effect on PTH transcription through the PTH promoter. To do this we expressed PTH mRNA driven by a viral promoter to express large amounts of PTH mRNA in HEK293 cells. Differences in PTH mRNA levels would therefore represent only post-transcriptional regulation. Expression of the CaR markedly decreased PTH mRNA levels and stability and conferred responsiveness to [Ca^2+^]_o _and the calcimimetic R568 in these cells. This was mediated by the PTH mRNA 3'-UTR ARE. Moreover, expression of the CaR in the HEK293 cells led to a shift from the interaction of the PTH mRNA with the stabilizing protein, AUF1, to the destabilizing protein, KSRP. Furthermore, the expression of CaR modified AUF1 post-translationally as previously shown *in vivo *[7,27]. Therefore, the expression of the CaR in this heterologous cell system reproduces the signal transduction that determines PTH gene expression in the parathyroid.

## Results

### The calcium receptor decreases parathyroid hormone mRNA levels post-transcriptionally in HEK293 cells

**Figure 1:**
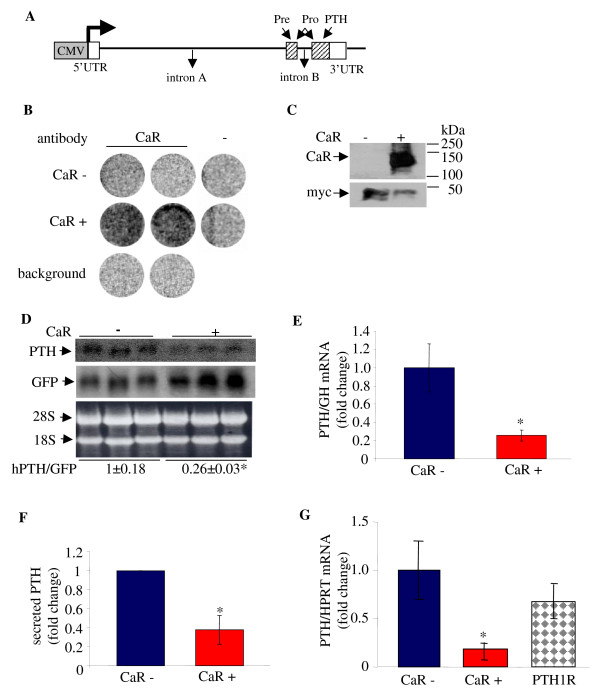
**Over-expression of the CaR decreases PTH mRNA levels in co-transfected HEK293 cells**. HEK293 cells were transiently co-transfected in triplicate with expression plasmids for the hPTH gene and control GFP or GH and either the CaR (CaR+) or empty vector (CaR-). **A**. Schematic representation of the human PTH expression plasmid used for transient transfections. The boxes show the CMV promoter (grey) and the PTH exons with the untranslated regions (UTRs) (white) and coding regions (diagonal lines). The arrows show the pre, pro and mature PTH. **B**. Immunohistochemistry on whole cells using an intact cell enzyme-linked immunoassay for the cell surface expression of the CaR. Untransfected (background), CaR (+) or control plasmid (-) transfected cells were analyzed using a CaR antibody or IgG (-). **C**. Immunoblot analysis of extracts from HEK293 cells co-transfected with expression plasmids for the CaR and myc-AUF1 as control plasmid, using anti-CaR or myc antibodies. **D-G**. Effect of CaR on PTH expression. **D**. Northern blot for hPTH and co-transfected GFP with the CaR (+) or an empty vector (-). Ethidium bromide staining of the membrane is shown as a loading control. **E**. qPCR for PTH and co-transfected GH mRNA levels from cells with (red) and without (blue) the CaR. **F**. Secreted PTH from cells as above 1 h after an incubation in fresh medium, 1 mM Ca^2+^. **G**. qRT-PCR for PTH mRNA levels from cells without and with either the CaR or the PTH1R (checkered). Data in D-G are expressed as fold change (mean ± SE) (n = 3). *, *P *< 0.01, CaR: control.

PTH gene expression is regulated post-transcriptionally by Ca^2+ ^and calcimimetics affecting PTH mRNA stability [7,15]. To focus on the effect of the CaR on PTH mRNA stability in a cell system we constructed an expression vector containing the full-length human PTH gene including its three exons and two introns (hPTH) driven by a viral SV40 (not shown) or CMV promoter (Figure 1A). Expression of PTH from both viral promoters gave similar results and we used the CMV promoter for further studies.

The hPTH expression plasmid was transiently transfected into HEK293 cells. The transcribed PTH mRNA was correctly spliced resulting in PTH mRNA of the expected size (Figure 1D). The PTH mRNA was translated into mature human PTH that was measured in the medium by radioimmunoassay (Figure 1F). We then studied the effect of the CaR on PTH mRNA levels in HEK293 cells expressing the PTH gene by transient transfection. The expression of the CaR was confirmed by immunohistochemistry on whole cells using an intact cell enzyme-linked immunoassay to determine cell surface expression [28] (Figure 1B) and by western blot (Figure 1C). Transfection efficiency was >95%, as indicated by fluorescent microscopy of the cells co-transfected with a green fluorescent protein (GFP) expression plasmid (not shown). Co-transfection of the human (h) CaR together with the hPTH plasmid resulted in a marked decrease in PTH mRNA levels by Northern blot (Figure 1D) and real time RT PCR (qPCR) (Figure 1E) corrected for co-transfected control genes GH (Figure 1E) or endogenous HPRT (Figure 1G). The decrease in PTH mRNA by the CaR was reflected in a decrease in secreted PTH at 48 h (Figure 1F). Transfection with another G protein coupled receptor (GPCR), the PTH receptor (PTH1R) had no effect on PTH mRNA levels, confirming the specificity of the CaR effect on PTH mRNA (Figure 1G).

### The calcium receptor decreases parathyroid hormone mRNA levels through the parathyroid hormone mRNA 3'-UTR AU-rich element

**Figure 2:**
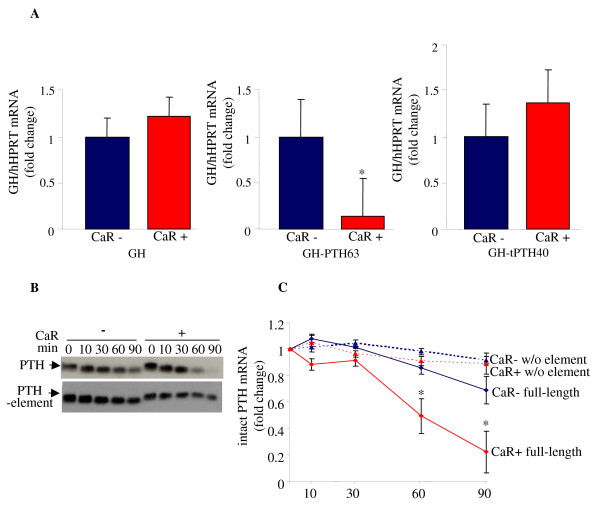
**The CaR decreases PTH mRNA levels and stability through the PTH mRNA 3'-UTR *cis *element**. **A**. Effect of CaR over-expression on reporter GH mRNA containing the PTH mRNA ARE. qRT-PCR for GH and control HPRT mRNA levels in cells transfected with either control (CaR-) or CaR (CaR+) and expression plasmids for GH (left panel), GH containing the PTH mRNA 63 nt ARE (GH-PTH63) (middle panel) or GH containing a 40 nt truncated ARE (GH-tPTH40) (right panel). Results are fold changes compared with cells without the CaR expressed as mean ± SE of three experiments. *, *P *< 0.05. **B**. Effect of CaR on PTH mRNA decay. Representative IVDA of transcripts for the full-length hPTH mRNA and the PTH mRNA with an internal deletion of the 3'-UTR ARE, incubated with extracts from cells expressing either the CaR or empty vector. **C**. Quantification of the amount of intact transcripts remaining with time related to time 0 (mean ± SE, in three repeat experiments; *, *P *< 0.05). Blue square, full length PTH mRNA without CaR (CaR-) and red square, with CaR (CaR+); blue triangle, PTH w/o ARE without CaR (CaR-) and red triangle, with the CaR, (CaR+).

We have previously reported that the regulation of PTH mRNA levels is dependent upon a *cis-*acting instability ARE in the PTH mRNA 3'-UTR [15,16,20]. We then determined if the regulation of PTH mRNA levels by the CaR is exerted through the PTH mRNA ARE, using a growth hormone (GH) reporter gene containing either the rat PTH 63 nt ARE (GH63) or a truncated non-functional PTH mRNA 40 nt element [29]. Over-expression of the CaR decreased GH63 mRNA levels but had no effect on wild-type GH mRNA levels or on a GH mRNA with the truncated 40 nt PTH mRNA ARE (Figure 2A). Our results indicate that the CaR specifically decreases PTH mRNA levels through the PTH mRNA ARE.

To study the effect of the CaR on PTH mRNA stability we used an *in vitro *degradation assay (IVDA) [16,20]. A radiolabeled polyadenylated *in vitro*-transcribed hPTH mRNA was incubated with extracts from cells transfected with either the CaR expression plasmid or a control plasmid. The amount of intact PTH mRNA remaining with time represents the decay of the transcript by the different extracts and is indicative of the decay rate *in vivo *[15,30]. The rate of PTH mRNA decay was increased by extracts from cells expressing the CaR compared with extracts from cells with control plasmid correlating with PTH mRNA levels in the transfected cells (Figure 2B, top gel and Figure 2C). We then performed IVDA using polyadenylated PTH mRNA with an internal deletion of the ARE (Figure 2B, bottom gel and Figure 2C). The PTH mRNA transcript lacking the ARE was stable throughout the experiment and in contrast to the full-length PTH mRNA, was not affected by expression of the CaR (Figure 2B and 2C). Our results indicate that the CaR specifically decreases steady-state PTH mRNA levels and PTH mRNA stability through the PTH mRNA ARE.

### Protein-PTH mRNA interactions by RNA immunoprecipitation assays

**Figure 3:**
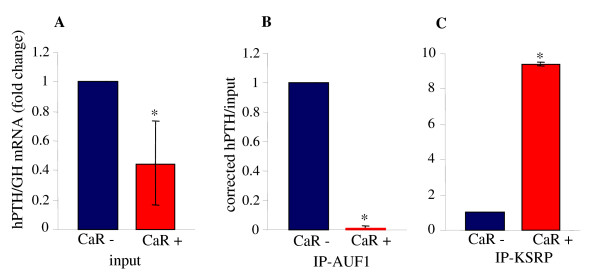
**Expression of the CaR in HEK293 cells decreases AUF1 and increases KSRP interaction with the PTH mRNA**. RNA immunoprecipitation (RIP) analysis of extracts from cells transiently transfected with expression plasmids for PTH and GH as control and either the CaR plasmid or empty vector. Immunoprecipitation was performed using antibodies for AUF1, KSRP or control IgG. Input **(A) **and immunoprecipitiated **(B, C) **samples were analyzed by qPCR for PTH and GH mRNA. Results are presented as PTH mRNA corrected for GH mRNA. PTH mRNA in the immunoprecipitated samples was corrected for PTH mRNA in the input. The results are mean ± SE of three repeat experiments. *, *P *< 0.05 compared with cells transfected with empty vector (CaR-).

*In vivo *in the rat parathyroid, PTH mRNA stability is determined by the regulated binding of AUF1 and KSRP to the PTH mRNA 3'-UTR [20]. To identify protein-mRNA interactions in the CaR transfected cells, we performed RNA immunoprecipitation (RIP) assays using extracts from HEK293 cells transfected with the CaR or control plasmids. Immunoprecipitation was performed with antibodies to AUF1, KSRP or control IgG followed by qRT PCR of the recovered RNA and input extracts. Parathyroid hormone mRNA was decreased in the CaR expressing extracts compared with control extracts (Figure 3A) as above (Figure 1D, 1E and 1G). The RIP assay showed that the amount of PTH mRNA bound to AUF1 was decreased in CaR-expressing cells compared with control cells (Figure 3B). In contrast, PTH mRNA bound to KSRP was increased in the CaR-expressing cells (Figure 3C). This binding pattern is consistent with the stabilizing function of AUF1 and the destabilizing function of KSRP on PTH mRNA [18,20]. The increased binding to KSRP and the decreased binding to AUF1 correlate with the lower levels of PTH mRNA induced by the CaR in the transfected cells.

### The calcium receptor leads to post-translational modification of the parathyroid hormone mRNA binding protein AUF1

**Figure 4:**
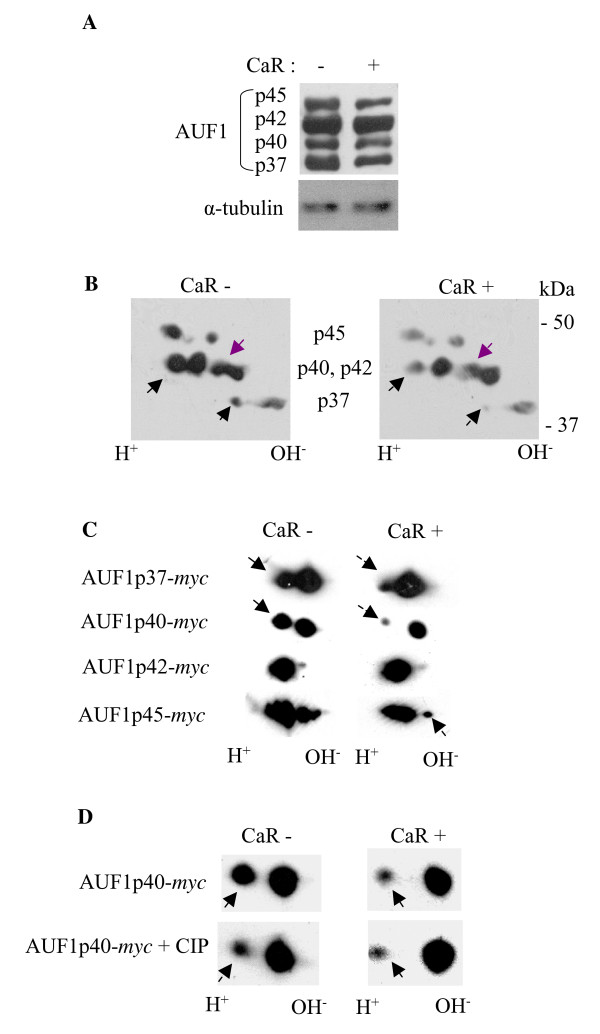
**AUF1 is post-translationally modified in HEK293 cells transfected with CaR**. **A**. 1D gels for AUF1 and α-tubulin as a loading control. **B**. 2D gel analysis for endogenous AUF1 in extracts from cells transiently transfected with the CaR (CaR+) plasmid or empty vector (CaR-). Molecular weight markers are shown on the right and the four AUF1 isoforms are indicated. **C**. 2D gel analysis of extracts from cells transiently transfected with the CaR plasmid or empty vector and the myc-AUF1 isoforms p37, p40, p42 or p45 separately. **D**. 2D analysis of extracts from cells transiently transfected with the CaR plasmid or empty vector and myc-AUF1p40 without and after treatment with a non-specific phosphatase (CIP). The results all represent one of two repeat experiments with similar results.

*In vivo *dietary-induced hypocalcemia and hypophosphatemia as well as adenine-induced renal failure and the calcimimetic R568 lead to post-translational modifications of AUF1 as shown by 2D gels [7,27]. To determine whether the effect of the CaR on PTH mRNA stability in the engineered cells involves post-translational modifications of AUF1 as it does in the parathyroid, we performed 2D gels on extracts from HEK293 cells transfected with the CaR expression plasmid or an empty vector as control. AUF1 has four isoforms of p37, p40, p42 and p45. There was no difference in AUF1 protein levels in the 1D gels between cells with and without the CaR, apart from a small decrease in p45 in the CaR-expressing cells (Figure 4A). However, 2D gels showed that endogenous AUF1 isoforms p37, p40 and p42 had a different distribution of the spots between extracts of cells with and without the CaR (Figure 4B). These results suggest that the CaR induces post-translational modifications in AUF1 in the transfected cells.

To analyze the four isoforms of AUF1 separately, we utilized myc-tagged AUF1 expression plasmids. HEK293 cells were transiently transfected with expression plasmids for myc- p37, p40, p42 or p45 isoforms of AUF1 together with the CaR or control plasmids. Cell extracts were analyzed on 2D gels with an anti-myc antibody. CaR expression led to changes in mobility of myc-AUF1 p37, p40 and also p45 but not p42 (Figure 4C). Unlike the changes in myc-AUF1 p45, endogenous AUF1 p45 was only slightly modified by CaR signaling. The change in endogenous p42 was not reflected in the myc-tagged transfected p42. The reasons for these discrepancies are not clear. AUF1 p40 undergoes reversible phosphorylation which may regulate ARE-directed mRNA turnover [31,32]. We therefore added a non-specific phosphatase to extracts from cells expressing myc-p40 with or without the CaR. Calf intestinal phosphatase (CIP) treatment of control extracts modified AUF1 to the form present in CaR-expressing extracts (Figure 4D). Treatment with CIP had no further effect on the CaR-expressing extracts. These results indicate that the CaR modifies AUF1 post-translationally and suggests that at least part of this change involves phosphorylation of isoform p40.

### Parathyroid hormone mRNA levels are regulated by [Ca^2+^]_o _and the calcimimetic R568 through the calcium receptor

**Figure 5:**
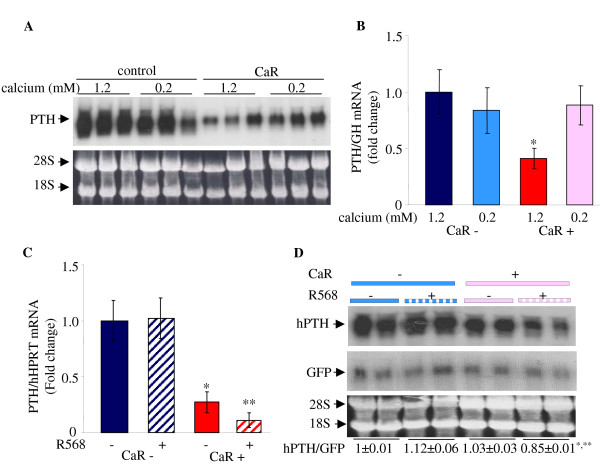
**Expression of the CaR decreases PTH mRNA levels and confers responsivity to a low [Ca^2+^]_o _and the calcimimetic R568**. **A**. Representative Northern blot analysis for PTH mRNA in cells transiently transfected in triplicate with hPTH and either the CaR plasmid or empty vector. After transfection cells were grown in 1.2 or 0.2 mM Ca^2+ ^medium for an additional 48 h. Ethidium bromide staining of the membrane is shown as loading control. **B**. qRT PCR for PTH corrected for co-transfected GH mRNAs for cells treated as in A. **C**. qPCR for PTH and HPRT mRNAs from cells expressing the hPTH gene and the CaR (CaR+) or empty vector (CaR-) in cells grown in 1.2 mM Ca^2+ ^supplemented with either R568 or vehicle. The results in A-C represent one of three repeat experiments performed in triplicate with similar results. **D**. Representative Northern blot for PTH and GFP mRNA levels in cells transfected with PTH and GFP and either CaR (CaR+) or empty vector (CaR-). The cells were grown in 0.2 mM calcium supplemented with R568 or vehicle. Quantification of the results is shown below the gel. The results are presented as mean ± SE of two repeat experiments performed in triplicate. *, *P *< 0.05, CaR+:CaR-; **, *P *< 0.05, CaR+, treated:CaR+, untreated.

We then studied the effect of a low-calcium medium on the regulation of PTH gene expression by the CaR in the transfected cells. HEK293 cells transiently co-transfected with expression plasmids for PTH and the CaR or a control plasmid were grown in a medium with either 0.2 or 1.2 mM Ca^2+^. Expression of the CaR decreased PTH mRNA in cells grown in physiological 1.2 mM Ca^2+ ^concentration by both Northern blots and qRT PCR (Figure 5A and 5B) as in Figure 1. Importantly, the low-Ca^2+ ^medium attenuated the decrease in PTH mRNA levels induced by the CaR (Figure 5A and 5B). There was no effect of a low-Ca^2+ ^medium in cells that did not express the CaR.

Calcimimetics are compounds that bind and activate the CaR [5]. We then performed the same experiments with and without the calcimimetic, R568 (Figure 4C). The CaR decreased PTH mRNA levels as before at 1.2 mM Ca^2+ ^(Figure 5C). Activation of the CaR by R568 decreased PTH mRNA levels at 1.2 mM Ca^2+ ^(Figure 5C) and also at 0.2 mM Ca^2+ ^(Figure 5D). 0.2 mM Ca^2+ ^prevented the decrease in PTH mRNA levels due to the CaR (Figure 5B and 5D). However, at 0.2 mM Ca^2+ ^the addition of R568 still effectively decreased PTH mRNA levels (Figure 5D). There was no effect of CaR expression on co-transfected GFP mRNA levels (Figure 5D) used as a control for transfection efficiency and loading. There was also no effect of R568 on PTH mRNA levels in cells co-transfected with the control plasmid that did not express the CaR (Figure 5C and 5D). The effect of R568 on PTH mRNA levels in the CaR-transfected cells at 0.2 mM Ca^2+ ^indicates that the calcimimetic is effective at activating the CaR even when the CaR is in a relaxed configuration. Therefore, in these engineered cells, the CaR is permissive for the effect of [Ca^2+^]_o _and R568 on PTH mRNA levels.

## Discussion

Primary bovine parathyroid cells in culture have been successfully used for the study of PTH secretion in the short term (hours) but not for longer term effects on PTH gene expression. This is because these primary monolayer cultures of parathyroid cells lose the expression of the CaR after 24 h in culture [33]. Parathyroid organoid cultures retain a prolonged calcium response and regulation of PTH mRNA stability by high [Ca^2+^]_o _and a calcimimetic [34,35]. However, there is no parathyroid cell line. Kifor et al [25] showed that the CaR activated the phosphotyrosine kinase in both CaR-transfected HEK293 cells and native bovine parathyroid cells. In CaR-expressing HEK293 cells, [Ca^2+^]_o _activates PI-PLC, PLA_2 _and PLD, similar to the effect in bovine primary parathyroid cell cultures [25,26,36]. There have been no studies on the effect of the CaR on the regulation of PTH gene expression using the transfected HEK293 cells. We therefore expressed the CaR together with the hPTH gene in HEK293 cells. The effect of [Ca^2+^]_o _and the calcimimetic R568 on PTH expression in the parathyroid *in vivo *is predominantly post-transcriptional, regulating PTH mRNA stability and not transcription through the PTH promoter [7,15]. This allowed us to use a viral promoter upstream of the hPTH gene to study the effect of the CaR signaling on PTH mRNA levels. The PTH gene was efficiently transcribed, processed and translated to mature immunoreactive PTH. Significantly, PTH mRNA and secreted PTH levels were decreased in cells expressing the CaR compared with control cells. The calcium concentration in the medium was 1.2 mM, which would activate the CaR to reduce PTH mRNA levels in the CaR-transfected cells. Therefore, the CaR suppresses PTH gene expression post-transcriptionally by acting on PTH mRNA driven by a viral and not the native PTH promoter. *In vitro *degradation assays using extracts from cells with or without the CaR showed that expression of the CaR led to a decrease in full-length PTH mRNA half-life but not the half-life of a PTH mRNA with an internal deletion of the PTH mRNA ARE. The decreased PTH mRNA stability correlated with decreased PTH mRNA steady state levels.

Dietary-induced changes in serum calcium and phosphate levels regulate PTH gene expression *in vivo *post-transcriptionally [14,15]. This regulation is mediated by the changes in binding of stabilizing *trans*-acting factors, AUF1 and Unr, and the destabilizing protein KSRP, to the defined *cis*-acting ARE in the PTH mRNA 3'-UTR [16,18-20]. The role of the PTH mRNA ARE in the regulation of PTH mRNA levels by the CaR was demonstrated by transfection experiments using a GH reporter mRNA containing the PTH mRNA 63 nt ARE. The CaR decreased GH-PTH ARE mRNA levels but had no effect on native GH mRNA or a GH mRNA containing a truncated PTH mRNA ARE. Calcium receptor-transfected HEK293 cells showed increased KSRP-PTH mRNA binding and decreased AUF1-PTH mRNA binding by RIP analysis, correlating with the decreased PTH mRNA levels and stability in the CaR-transfected cells. Therefore, the signaling of the CaR in the parathyroid is maintained in the transfected cells as reflected in changes in AUF1 and KSRP PTH mRNA interactions and the role of the PTH mRNA ARE in this regulation.

AUF1-PTH mRNA binding is increased by hypocalcemia or chronic kidney disease, or decreased by hypophosphatemia or administration of the calcimimetic R568 [7,15]. These changes in serum Ca^2+ ^or phosphate and kidney disease are associated with post-translational modifications of AUF1. AUF1 isoforms p40 and p42 are modified by dietary-induced hypocalcemia, and the secondary hyperparathyroidism of chronic kidney disease and its treatment by R568 [7,27]. We now show that expression of the CaR in the engineered cells also induces post-translational modifications in both endogenous AUF1 and myc-tagged transfected AUF1 isoforms that are similar to the modifications *in vivo *in the parathyroid. The modification of at least AUF1 p40 involves phosphorylation where expression of the CaR is associated with dephosphorylation. Post-translational modifications of this isoform, AUF1 p40, are altered concomitant with changes in RNA binding activity and stabilization of ARE-containing mRNAs in phorbol ester-treated monocytic leukemia cells [32]. AUF1 p40 recovered from polysomes was phosphorylated on Ser83 and Ser87 in untreated cells but lost these modifications following phorbol ester treatment. It was suggested that selected signal transduction pathways may regulate ARE-directed mRNA turnover by reversible phosphorylation of AUF1 p40 [31,32]. Therefore, both in the CaR-transfected cells and in the phorbol ester-treated cells, dephosphorylation of AUF1 p40, is associated with decreased AUF1 activity. In the case of PTH mRNA, inactivity of AUF1 results in a less stable PTH mRNA after expression of the CaR, as AUF1 is a PTH mRNA stabilizing protein [18,27].

We also show that expression of the CaR conferred responsiveness to low [Ca^2+^]_o _and the calcimimetic R568, underlining the relevance of this cellular model to the parathyroid. A low- [Ca^2+^]_o _medium increased PTH expression only in the cells expressing the CaR. Similarly, R568 decreased PTH expression through the CaR at both 1.2 and 0.2 mM [Ca^2+^]_o_. Therefore, regulation of PTH mRNA levels by expression of the CaR itself and both its activation by the calcimimetic and its relaxation by a low [Ca^2+^]_o _are maintained in these transfected cells. This is the first demonstration that the CaR can regulate PTH gene expression in heterologous cells. This regulation is obtained through the post-transcriptional mechanisms that involve changes in PTH mRNA-protein interactions in the parathyroid (Table 1). It is likely that PTH secretion would exhibit a similar regulation by calcium as PTH mRNA levels, given its dependence on the latter. However, secretion was not measured as a function of the extracellular calcium concentration in the heterologous HEK293 cell system, in which there is a prominent component of constitutive secretion, unlike the parathyroid cell [37,38]. Secretion in HEK293 cells may be independently regulated by the CaR although we have not studied this question. The parathyroid cell is unique in that the stimulus for secretion, gene expression and cell proliferation is a low [Ca^2+^]_o _and not a high [Ca^2+^]_o_. Inverse control of secretion by a low [Ca^2+^]_o _of PTHrP secretion also occurs in the epithelial cells of the lactating breast and certain breast cell lines [39,40]. In addition high [Ca^2+^]_o _decreases hormone secretion in the secretion of renin by renal juxtaglomerular cells [41]. Calcium receptor activation increases cell proliferation in cultured cells [42], but inhibits parathyroid proliferation *in vivo *[43]. It is remarkable and intriguing that despite these differences, the transfected cell system retains so many of the native characteristics of the unique parathyroid cell, specifically the relationship between calcium and PTH mRNA stability. The CaR-mediated decrease in PTH expression in these cells mirrors the marked increase in serum PTH *in vivo *in mice and men with inactivating mutations of the CaR [6,10,36,43,44]. These results support the concept that the parathyroid cell is geared to constitutively synthesize and secrete PTH, and it is the presence of a functioning CaR that tonically inhibits PTH expression and secretion and allows responsivity to [Ca^2+^]_o_.

**Table 1:** Regulation of PTH gene expression in HEK293 cells transfected with the CaR and hPTH plasmids and in the rat parathyroid by calcium

	HEK293 cells + CaR	Rat parathyroid
PTH transcription	not applicable	no effect

PTH mRNA levels	regulated	regulated

PTH mRNA stability	regulated	regulated

PTH secretion	unknown	regulated

AUF1 and KSRP-PTH mRNA interactions	regulated	regulated

AUF1 protein modifications	regulated	regulated

regulation by [Ca^2+^]_o _and R568	regulated	regulated

AUF1 = AU rich binding factor; KSRP = KH domain splicing regulatory protein; PTH = parathyroid hormone

## Conclusion

The expression of the CaR in transfected HEK293 cells decreases PTH gene expression post-transcriptionally and is sufficient to confer the regulation of PTH gene expression by [Ca^2+^]_o _and a calcimimetic, R568. The CaR decreases PTH gene expression in these engineered cells through the balanced interactions of the *trans*-acting factors KSRP and AUF1 with PTH mRNA and the PTH mRNA ARE, as *in vivo *in the parathyroid. This is the first demonstration that the CaR can regulate PTH gene expression in heterologous transiently transfected cells.

## Methods

### Cells and transfection

HEK293 cells were transiently co-transfected in a 10 cm dish with 5 μg expression plasmids for the PTH gene and the CaR plasmid or a control plasmid using a Calcium Phosphate Transfection Kit (Sigma, St Louis, MO, USA). At 48 h the cells expressing the CaR or controls were detached and replated in 24 well plates and treated as indicated, in triplicates for 48 h. Cells were grown in DMEM supplemented with 10% Fetal Bovine Serum (Biological Industries, Beit Haemek, Israel), L-Glutamine (Biological Industries, Beit Haemek, Israel) and Pen-Strep solution (Biological Industries, Beit Haemek, Israel). Calcium concentration in the medium was 1.2 mM. In some experiments Ca^2+^-free DMEM medium was used (0.2 mM Ca^2+^) and CaCl_2 _was added as indicated. R568, a gift from Amgen (Thousand Oaks, CA, USA) was dissolved in DDW and added to the medium at a final concentration of 1 μM.

### RNA extraction and analysis

RNA extraction was performed using Tri reagent (Molecular Research Center, Cincinnati, OH, USA). mRNA concentration was determined using a NanoDrop ND-1000 spectrophotometer (NanoDrop Technologies; Wilmington, DE, USA). For real-time PCR 1 μg mRNA was used as template for RT PCR with Maxime RT PreMix (Random primer) from iNtRON (Gyeonggi-do, Korea). qPCR was performed using SYBR Green ROX Mix (ABgene, Surrey, UK) according to the manufacturer's instructions in 7900HT Fast Real-Time PCR Systems from Applied Biosystems.

The following primers were used: hPTH (5'-gggtctgcagtccaattcat-3' and 5'-cagatttcccatccgatttt-3'), hGH (5'-gggaggctggaagatggc-3' and 5'-cgttgtgtgagtttgtgtcgaac-3'), hHPRT (5' tttgaatcatgtttgtgtcattagtga and 5' ttccaaactcaacttgaactctcatc). For Northern analysis RNA was run on agarose formaldehyde gels and blotted using a Hybond-N membrane (Amersham Biosciences, Little Chalfont, UK). Membranes were hybridized to fragments for hPTH or GFP [20,29]. In some experiments ethidium bromide staining of membranes was used for quantification of 18S or 28S as loading controls.

### Plasmids

For the hPTH expression plasmid, a *Hpa*II fragment including the three exons and two introns of the hPTH gene from plasmid pPTHg108 [20] was inserted downstream of the CMV promoter in pcDNA3 expression plasmid (Invitrogen, San Diego, CA, USA). The hCaR plasmid was kindly provided by M Lohse (Wurzburg, Germany), the GFP pEGFP-C1 (Clontech, Palo Alto, CA, USA) and hGH (kindly provided by O Meyuhas, Jerusalem, Israel [45]) were used as controls. The GH-PTH63 or GH-tPTH40 expression plasmids contained 63 bp or a truncated 40 bp fragments of the PTH mRNA 3'-UTR ARE that were inserted between the coding region and the 3'-UTR of GH mRNA [29]. hPTH1R was provided by MA Levine, Cleveland, OH, USA). In some experiments cells co-transfected with the GFP plasmid were analyzed by fluorescent microscopy to estimate transfection efficiency, which was always >90%.

### Immunoreactive parathyroid hormone

Medium was replaced 1 h prior to collection and analyzed using the Immulite 2000 Intact PTH assay (Los Angeles, CA, USA)

### In Vitro Degradation Assays

Radiolabeled transcripts (200,000 cpm) were incubated with 40 μg protein extract from cells in a volume of 50 μl and in a reaction buffer containing 3 mM Tris HCl, pH 7.5, 2 mM MgCl_2_, 3 mM NaCl, 10 mM ATP and 30 units RNasin. At timed intervals samples were removed, RNA extracted, separated on agarose gels and analyzed by autoradiography as described [15].

The following plasmids were used as templates for RNA transcription: pBluescript II KS plasmid containing either the full-length rat PTH cDNA (772 bp) or a PTH cDNA with an internal deletion of the PTH mRNA ARE and a stretch of ~150 dT nucleotides that by *in vitro *transcription produced a poly(A) tail. The plasmids were linearized with *Sma*I [20].

### 2D gel electrophoresis

Analysis was performed as previously described [27].

### RNA immunoprecipitation

Transiently transfected cells were grown in a 14 cm dish for 48 h, collected in ice-cold PBS, pelleted and re-suspended in RIPA buffer (containing 150 mM NaCl, 1% NP40, 0.5% sodium deoxycholate, 0.1% SDS and protease inhibitors) supplemented with RNase inhibitors (Promega, Madison, WI, USA) and homogenized by pipetting. Equal amounts of whole cell extracts were immunoprecipitated with Protein A agarose-bound anti-KSRP or AUF1 antibody beads (Calbiochem, Darmstadt, Germany) or IgG as control after incubation for 2 hr at 4°C. The beads were then washed with modified RIPA buffer (supplemented with 1 M NaCl, 1% Sodium Deoxycholate, 1 mM EDTA and 2 M urea). RNA was extracted and analyzed by qPCR for PTH and GH mRNA using SYBR Green ROX Mix as described above.

### Intact cell enzyme-linked immunoassay

The assay was performed as previously described [27]. In brief: 48 h after transfection cells were detached in PBS supplemented with BSA and EDTA. The cells were then pelleted and incubated for 1 h at 4°C with the anti-CaR antibody followed by PBS wash (× 3) and incubation with the secondary HRP-conjugated antibody. Cells were then washed (× 3) prior to adding the HRP substrate and the fluorescent signal was visualized using an electrochemiluminescent reader.

### Antibodies

Anti-KSRP was a gift from R Gherzi, Genoa, Italy. Anti-AUF was from Upstate (Charlottesville, VA, USA). Anti-*myc *was from Cell Signaling (Boston, MA, USA). Anti-CaR was from Abcam (Cambridge, UK).

### Statistics

Statistical analysis was performed using Microsoft Excel (Microsoft Corporation, Redmond, WA, USA). Values are reported as mean ± SEM unless stated otherwise. A 2-tailed Student's *T*-test was used to assess differences from the control group. A *P *value of less than 0.05 was considered significant.

## Abbreviations

ARE: AU-rich element; AUF1: AU rich binding factor; CaR: calcium receptor; CIP: calf intestinal phosphatase; GFP: green fluorescent protein; HEK: human embryonic kidney; HPT: hyperparathyroidism; IVDA: *in vitro *degradation assay; KSRP: KH domain splicing regulatory protein; PTH: parathyroid hormone; qRT PCR: quantitative reverse transcriptase polymerase chain reaction; RIP: RNA immunoprecipitation; TM: transmembrane; UTR: untranslated region.

## Authors' contributions

Most experiments were performed by HG who also conceived and designed the experiments. VL-M performed the experiments in Fig 4C–D and MN and TM assisted in different experiments. JS and TN-M conceived, designed and wrote the manuscript. All authors read and approved the final manuscript.

## References

[B1] Brown EM, Gamba G, Riccardi D, Lombardi M, Butters R, Kifor O, Sun A, Hediger MA, Lytton J, Hebert J (1993). Cloning and characterization of an extracellular Ca^2+^-sensing receptor from bovine parathyroid. Nature.

[B2] Silver J, Naveh-Many T, Kronenberg HM, Bilezikian JB, Raisz LG, Rodan GA (2002). Parathyroid hormone: molecular biology. Principles of bone biology.

[B3] Rodriguez M, Nemeth E, Martin D (2005). The calcium-sensing receptor: a key factor in the pathogenesis of secondary hyperparathyroidism. Am J Physiol Renal Physiol.

[B4] Hofer AM, Brown EM (2003). Extracellular calcium sensing and signalling. Nat Rev Mol Cell Biol.

[B5] Nemeth EF, Steffey ME, Hammerland LG, Hung BC, Van Wagenen BC, DelMar EG, Balandrin MF (1998). Calcimimetics with potent and selective activity on the parathyroid calcium receptor. Proc Natl Acad Sci USA.

[B6] Hu J, Spiegel AM (2003). Naturally occurring mutations of the extracellular Ca2+-sensing receptor: implications for its structure and function. Trends Endocrinol Metab.

[B7] Levi R, Ben Dov IZ, Lavi-Moshayoff V, Dinur M, Martin D, Naveh-Many T, Silver J (2006). Increased parathyroid hormone gene expression in secondary hyperparathyroidism of experimental uremia is reversed by calcimimetics: correlation with posttranslational modification of the trans acting factor AUF1. J Am Soc Nephrol.

[B8] Colloton M, Shatzen E, Miller G, Stehman-Breen C, Wada M, Lacey D, Martin D (2005). Cinacalcet HCl attenuates parathyroid hyperplasia in a rat model of secondary hyperparathyroidism. Kidney Int.

[B9] Wettschureck N, Lee E, Libutti SK, Offermanns S, Robey PG, Spiegel AM (2007). Parathyroid-specific double knockout of Gq and G11 alpha-subunits leads to a phenotype resembling germline knockout of the extracellular Ca2+ -sensing receptor. Mol Endocrinol.

[B10] Ho C, Conner DA, Pollak MR, Ladd DJ, Kifor O, Warren HB, Brown EM, Seidman JG, Seidman CE (1995). A mouse model of human familial hypocalciuric hypercalcemia and neonatal severe hyperparathyroidism. Nat Genet.

[B11] Kos CH, Karaplis AC, Peng JB, Hediger MA, Goltzman D, Mohammad KS, Guise TA, Pollak MR (2003). The calcium-sensing receptor is required for normal calcium homeostasis independent of parathyroid hormone. J Clin Invest.

[B12] Tu Q, Pi M, Karsenty G, Simpson L, Liu S, Quarles LD (2003). Rescue of the skeletal phenotype in CasR-deficient mice by transfer onto the Gcm2 null background. J Clin Invest.

[B13] Silver J, Kilav R, Naveh-Many T (2002). Mechanisms of secondary hyperparathyroidism. Am J Physiol Renal Physiol.

[B14] Kilav R, Silver J, Naveh-Many T (1995). Parathyroid hormone gene expression in hypophosphatemic rats. J Clin Invest.

[B15] Moallem E, Silver J, Kilav R, Naveh-Many T (1998). RNA protein binding and post-transcriptional regulation of PTH gene expression by calcium and phosphate. J Biol Chem.

[B16] Kilav R, Silver J, Naveh-Many T (2001). A conserved cis-acting element in the parathyroid hormone 3'-untranslated region is sufficient for regulation of RNA stability by calcium and phosphate. J Biol Chem.

[B17] Bell O, Silver J, Naveh-Many T (2005). Identification and characterization of *cis *-acting elements in the human and bovine parathyroid hormone mRNA 3'-untranslated region. J Bone Miner Res.

[B18] Sela-Brown A, Silver J, Brewer G, Naveh-Many T (2000). Identification of AUF1 as a parathyroid hormone mRNA 3'-untranslated region binding protein that determines parathyroid hormone mRNA stability. J Biol Chem.

[B19] Dinur M, Kilav R, Sela-Brown A, Jacquemin-Sablon H, Naveh-Many T (2006). *In vitro *evidence that upstream of N-ras participates in the regulation of parathyroid hormone messenger ribonucleic acid stability. Mol Endocrinol.

[B20] Nechama M, Ben Dov IZ, Briata P, Gherzi R, Naveh-Many T (2008). The mRNA decay promoting factor K-homology splicing regulator protein post-transcriptionally determines parathyroid hormone mRNA levels. FASEB J.

[B21] Brown EM, Gardner DG, Aurbach GD (1980). Effects of the calcium ionophore A23187 on dispersed bovine parathyroid cells. Endocrinology.

[B22] Almaden Y, Canalejo A, Hernandez A, Ballesteros E, Garcia-Navarro S, Torres A, Rodriguez M (1996). Direct effect of phosphorus on parathyroid hormone secretion from whole rat parathyroid glands in vitro. J Bone Miner Res.

[B23] Silver J, Russell J, Sherwood LM (1985). Regulation by vitamin D metabolites of messenger ribonucleic acid for preproparathyroid hormone in isolated bovine parathyroid cells. Proc Natl Acad Sci USA.

[B24] Moallem E, Silver J, Naveh-Many T (1995). Regulation of parathyroid hormone messenger RNA levels by protein kinase A and C in bovine parathyroid cells. J Bone Miner Res.

[B25] Kifor O, Diaz R, Butters R, Brown EM (1997). The Ca2+-sensing receptor (CaR) activates phospholipases C, A2, and D in bovine parathyroid and CaR-transfected, human embryonic kidney (HEK293) cells. J Bone Miner Res.

[B26] Kifor O, MacLeod RJ, Diaz R, Bai M, Yamaguchi T, Yao T, Kifor I, Brown EM (2001). Regulation of MAP kinase by calcium-sensing receptor in bovine parathyroid and CaR-transfected HEK293 cells. Am J Physiol Renal Physiol.

[B27] Bell O, Gaberman E, Kilav R, Levi R, Cox KB, Molkentin JD, Silver J, Naveh-Many T (2005). The protein phosphatase calcineurin determines basal parathyroid hormone gene expression. Mol Endocrinol.

[B28] Ray K, Fan GF, Goldsmith PK, Spiegel AM (1997). The carboxyl terminus of the human calcium receptor. Requirements for cell-surface expression and signal transduction. J Biol Chem.

[B29] Kilav R, Bell O, Le SY, Silver J, Naveh-Many T (2004). The parathyroid hormone mRNA 3'-untranslated region AU-rich element is an unstructured functional element. J Biol Chem.

[B30] Fritz DT, Ford LP, Wilusz J (2000). An *in vitro *assay to study regulated mRNA stability. Sci STKE.

[B31] Wilson GM, Lu J, Sutphen K, Sun Y, Huynh Y, Brewer G (2003). Regulation of A + U-rich element-directed mRNA turnover involving reversible phosphorylation of AUF1. J Biol Chem.

[B32] Wilson GM, Lu J, Sutphen K, Suarez Y, Sinha S, Brewer B, Villanueva-Feliciano EC, Ysla RM, Charles S, Brewer G (2003). Phosphorylation of p40AUF1 regulates binding to A + U-rich mRNA-destabilizing elements and protein-induced changes in ribonucleoprotein structure. J Biol Chem.

[B33] Brown AJ, Zhong M, Ritter C, Brown EM, Slatopolsky E (1995). Loss of calcium responsiveness in cultured bovine parathyroid cells is associated with decreased calcium receptor expression. Biochem Biophys Res Commun.

[B34] Ritter CS, Slatopolsky E, Santoro S, Brown AJ (2004). Parathyroid cells cultured in collagen matrix retain calcium responsiveness: importance of three-dimensional tissue architecture. J Bone Miner Res.

[B35] Ritter CS, Pande S, Krits I, Slatopolsky E, Brown AJ (2008). Destabilization of parathyroid hormone mRNA by extracellular Ca2+ and the calcimimetic R-568 in parathyroid cells: role of cytosolic Ca and requirement for gene transcription. J Mol Endocrinol.

[B36] Pearce SH, Bai M, Quinn SJ, Kifor O, Brown EM, Thakker RV (1996). Functional characterization of calcium-sensing receptor mutations expressed in human embryonic kidney cells. J Clin Invest.

[B37] Groskreutz DJ, Sliwkowski MX, Gorman CM (1994). Genetically engineered proinsulin constitutively processed and secreted as mature, active insulin. J Biol Chem.

[B38] Beuret N, Stettler H, Renold A, Rutishauser J, Spiess M (2004). Expression of regulated secretory proteins is sufficient to generate granule-like structures in constitutively secreting cells. J Biol Chem.

[B39] Mamillapalli R, VanHouten J, Zawalich W, Wysolmerski J (2008). Switching of G-protein usage by the calcium-sensing receptor reverses its effect on parathyroid hormone-related protein secretion in normal versus malignant breast cells. J Biol Chem.

[B40] VanHouten J, Dann P, McGeoch G, Brown EM, Krapcho K, Neville M, Wysolmerski JJ (2004). The calcium-sensing receptor regulates mammary gland parathyroid hormone-related protein production and calcium transport. J Clin Invest.

[B41] Ortiz-Capisano MC, Ortiz PA, Garvin JL, Harding P, Beierwaltes WH (2007). Expression and function of the calcium-sensing receptor in juxtaglomerular cells. Hypertension.

[B42] McNeil SE, Hobson SA, Nipper V, Rodland KD (1998). Functional calcium-sensing receptors in rat fibroblasts are required for activation of SRC kinase and mitogen-activated protein kinase in response to extracellular calcium. J Biol Chem.

[B43] Pollack MR, Brown EM, Chou Y-HW, Hebert SC, Marx SJ, Steinman B, Levi T, Seidman CE, Seidman JG (1993). Mutations in the human Ca^2+ ^-sensing receptor gene cause familial hypocalciuric hypercalcemia and neonatal severe hyperparathyroidism. Cell.

[B44] Pearce SH, Williamson C, Kifor O, Bai M, Coulthard MG, Davies M, Lewis-Barned N, McCredie D, Powell H, Kendall-Taylor P, Brown EM, Thakker RV (1996). A familial syndrome of hypocalcemia with hypercalciuria due to mutations in the calcium-sensing receptor [see comments]. N Engl J Med.

[B45] Levy S, Avni D, Hariharan N, Perry RP, Meyuhas O (1991). Oligopyrimidine tract at the 5' end of mammalian ribosomal protein mRNAs is required for their translational control. Proc Natl Acad Sci USA.

